# Improved technique of personalised surgical guides generation for mandibular free flap reconstruction using an open-source tool

**DOI:** 10.1186/s41747-021-00229-x

**Published:** 2021-07-28

**Authors:** Luka Šimić, Vjekoslav Kopačin, Ivan Mumlek, Josip Butković, Vedran Zubčić

**Affiliations:** 1 Faculty of Electrical Engineering, Computer Science and Information Technology Osijek, Josip Juraj Strossmayer University of Osijek, Kneza Trpimira 2B, HR-31000 Osijek, Croatia; 2 Department of Diagnostic and Interventional Radiology, University Hospital Osijek, Josipa Huttlera 4, HR-31000 Osijek, Croatia; 3 Faculty of Medicine, Department of Biophysics and Radiology, Josip Juraj Strossmayer University of Osijek, Josipa Huttlera 4, HR-31000 Osijek, Croatia; 4 Department of Maxillofacial Surgery, University Hospital Osijek, Josipa Huttlera 4, HR-31000 Osijek, Croatia; 5 Faculty of Medicine, Department of Otorhinolaryngology and Maxillofacial Surgery, Josip Juraj Strossmayer University of Osijek, Josipa Huttlera 4, HR-31000 Osijek, Croatia

**Keywords:** Computer-aided design, Mandibular reconstruction, Medical informatics applications, Printing (three-dimensional), Surgery (computer-assisted)

## Abstract

With advancements in computer systems, computer graphics and medical imaging technologies, clinicians strive for a personalised approach to patient treatment. Therefore, the production of personalised surgical guides is becoming standard. While proprietary software solutions for mandibular reconstruction planning exist, they are often not available due to their high costs. There are multiple alternative methods available, which utilise open-source technologies and free software, but they use advanced three-dimensional (3D) computer-aided design (CAD) concepts. The goal of this article is to provide end-users (surgeons, radiologists, or radiology technicians) with a tool that offers an intuitive interface and a simple workflow. The tool provides only the necessary methods offering a high degree of automation and abstracting the underlying 3D CAD concepts. This is accomplished by providing an add-on (written in Python) for a free and open-source software package Blender.

## Key points

- An intuitive add-on for open-source software Blender enables a reliable method for fast and accurate personalised surgical guide generation and visualisation.
- Advanced computer-aided design concepts were abstracted behind a simplified interface.
- An open-source tool brings expensive technology closer to medical institutions and healthcare professionals with lower budgets, thus improving surgery outcomes and reducing overall healthcare costs.

## Background

The osteocutaneous free flap was first described by Taylor and his co-workers to treat a defect of the mandible in 1979 [1]. It became standard for complex oromandibular reconstruction following resection of tumours, as well as osteomyelitic and osteonecrotic parts of the mandible [1]. The goal of this technique is to achieve both functional and aesthetic restoration of the lower jaw.

Conventional approaches to mandibular reconstruction can be long and exhausting for surgical teams, as well as imprecise [2]. Numerous articles describe how beneficial virtual surgical planning and the use of three-dimensional (3D) printed models and surgical cutting guides could be to reduce surgery time [3–8]. Dell’Aversana Orabona et al. [7] showed that low-cost, 3D printed, “in-hospital” surgical guides have equally good post-op results as using commercially available computer-aided design (CAD)/computer-aided manufacturing (CAM) software or cutting guides. Ganry et al. [8] described a technique for surgical cutting guide generation using the open-source software Blender (Blender Foundation, Amsterdam, The Netherlands).

Production of surgical cutting guides still mainly lies in the domain of commercial biomechanical engineering companies whose service needs to be outsourced and can be expensive. Therefore, the described technique allowed hospitals to reduce costs. According to Ganry et al. [8], first, osteotomy planes on the mandible are positioned and represent the direction/location of the saw during the osteotomy. Then, mandible osteotomy guides are generated using mesh modelling techniques and a set of Boolean operations. An armature object and additional osteotomy planes for the fibula are then created, with their number depending on the required number of osteotomies. Fibula osteotomy planes must be connected to the armature object using a process called parenting. Again, using mesh modelling and Boolean operations, fibula guides are created [8]. These steps require the end-users to be proficient in the use of 3D CAD/CAM software in order to produce good results consistently. Numajiri et al. [9] described their process for fibula guide generation using Blender and according to them; the process lasts under 50 min. In comparison to Ganry et al.’s method [8], the process was simplified but still mostly manual and still requires advanced knowledge of Blender for 3D modelling [9].

The method we describe herein is loosely based on the technique described by Ganry et al. [8]. It is provided in the form of an add-on for the Blender software package. The software performed well on multiple systems, ranging from high-end desktop personal computers to mid-range laptop computers.

## Data acquisition and 3D model generation

Good planning and appropriate “Digital Imaging and Communications in Medicine” (DICOM) data acquisition are necessary to generate anatomically accurate 3D models. Since most patients who are considered to undergo the above-mentioned procedure are the ones with malignant disease, contrast-enhanced computed tomography (CT) scanning of the neck region is performed. Sometimes the thorax is included as part of tumour staging. Slice thickness ranging from 0.6 to 1 mm is appropriate, as lower resolution scans might result in the loss of anatomical details [10].

**Fig. 1:**
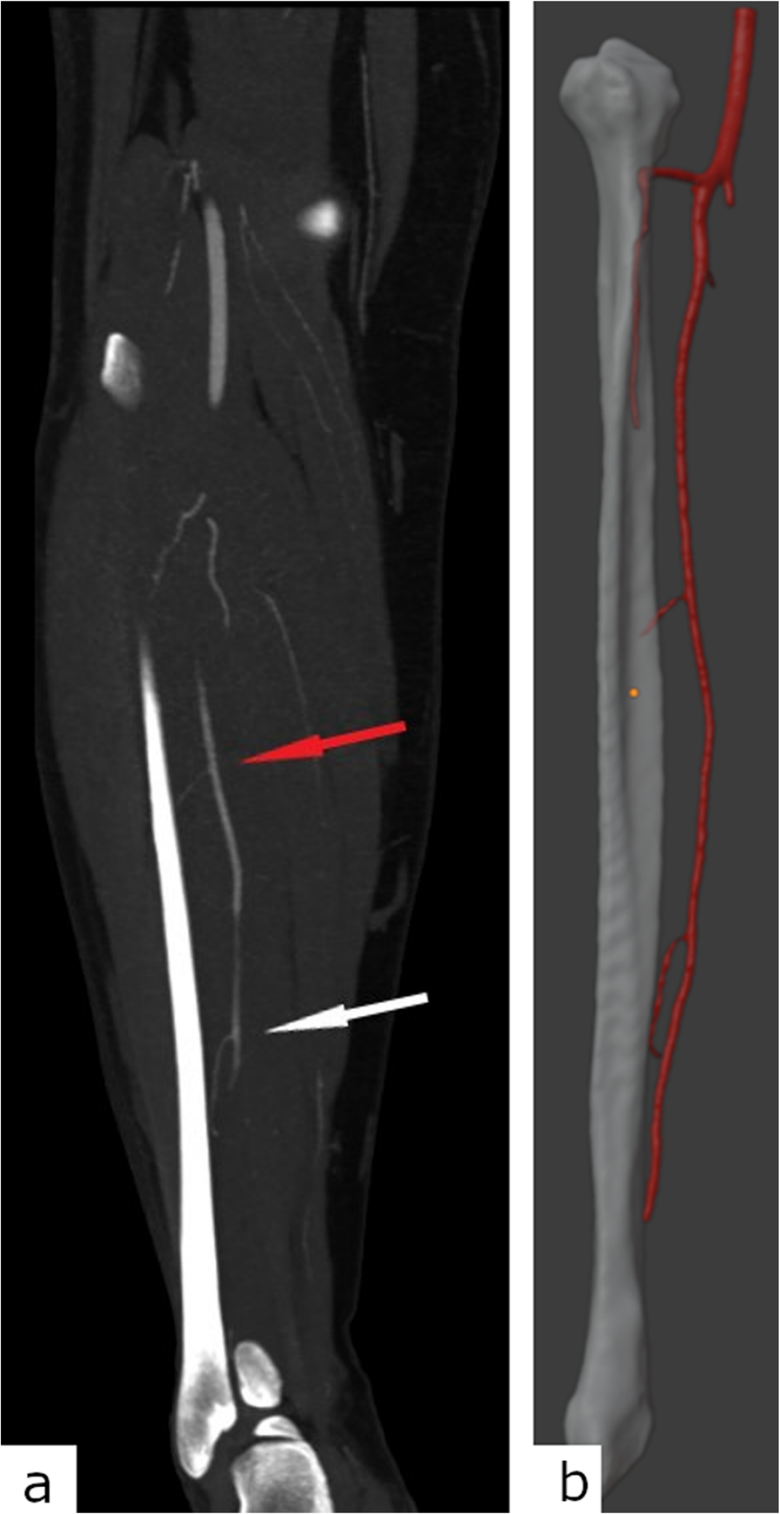
Visualisation of the peroneal artery perforators for the appropriate fibula orientation in space. **a** Coronal maximum intensity projection thick reconstructions of the right lower leg computed tomography angiography shows a good delineation of the proximal (red arrow) and distal (white arrow) peroneal artery perforators. **b** Matching three-dimensional model of the same patient’s right fibula and peroneal artery with visible perforators

To produce the guides, a 3D model of the fibula is needed as well. CT scanning of both lower legs should be performed. Again, slice thickness from 0.6 to 1 mm yields appropriate results. Some authors propose CT angiography of lower legs so the fibula could be appropriately oriented in space during the 3D modelling phase (Fig. 1). The fibular artery should face the oral side of the reconstructed mandibular arch for easier and more successful anastomosis [8].

Keeping in mind the above-mentioned requirements for a good quality 3D model, the following CT scanning protocols of the neck region and CT angiography of the lower legs proved satisfactory. Patients are examined using a multidetector CT scanner (Somatom Definition AS, Siemens Healthineers). The scanning region is from the aortic arch to the skull base, including the whole mandible. Scanning parameters are as follows: collimation, 128 × 0.6 mm; slice thickness, 3 mm; pitch, 0.6; gantry rotation time, 0.5 s; kVp, 120; mAs, 200; and craniocaudal scanning direction. The split bolus technique is used for intravenous application of contrast agent: using an automated power injector, 40 mL of nonionic, iodinated contrast agent is applied at 3 mL/s flow rate, followed by 20 mL of saline solution at the same flow rate. After 40 s from the first bolus application, a second bolus of contrast medium is applied with the same parameters. The bolus-tracking technique with the circular region of interest (ROI) is placed in the aortic arch, and the auto-trigger threshold value is set at 100 HU. Using both soft and bone kernels for the image reconstruction, slices with a thickness ranging from 0.625 to 1 mm should be sent to the “picture archiving and communication system” (PACS). If available, metal artefact reduction algorithms should be applied in patients with metal prostheses or amalgam tooth fillings.

Then, CTA of the lower legs is performed. The patient is oriented feet first, and the scanning region should include the region from the patellar apex, down to the calcaneus, including both fibulas completely. Scanning parameters are as follows: collimation, 128 × 0.6mm; slice thickness, 1 mm; pitch, 0.5; gantry rotation time, 0.5 s; kVp, 120; mAs, 120; and craniocaudal scanning direction. Using a power injector, 60 mL of nonionic iodinated contrast agent is applied with high flow rates (4.5−5 mL/s). The bolus-tracking technique is used with a ROI placed on the popliteal artery, and the auto-trigger value is set to 120 HU. If the ROI was misplaced or the patient moved between scout and contrast application, scanning could be started manually when the arterial lumen is adequately opacified with the contrast blood. Alternative, but equally effective approach is to use the “test bolus” technique. Thin slice reconstructions (ranging from 0.6 to 1 mm) are sent to PACS.

The obtained DICOM data is imported into any commercial or freeware software capable of image segmentation and generation of 3D meshes from segmented images, such as 3D Slicer [11]. In younger patients with preserved cortical bone mass and without atherosclerotic changes of the artery walls, it is possible to perform automatic segmentation of desired structures. In older patients with osteopenia and atherosclerotic changes, semi-automatic segmentation is performed for sufficient results. The resulting mesh can then be exported into any supported format, commonly “STL”: stereolithography interface format; standard triangulation language; or standard tessellation language.

## Generating surgical guides using the Add-On

**Fig. 2:**
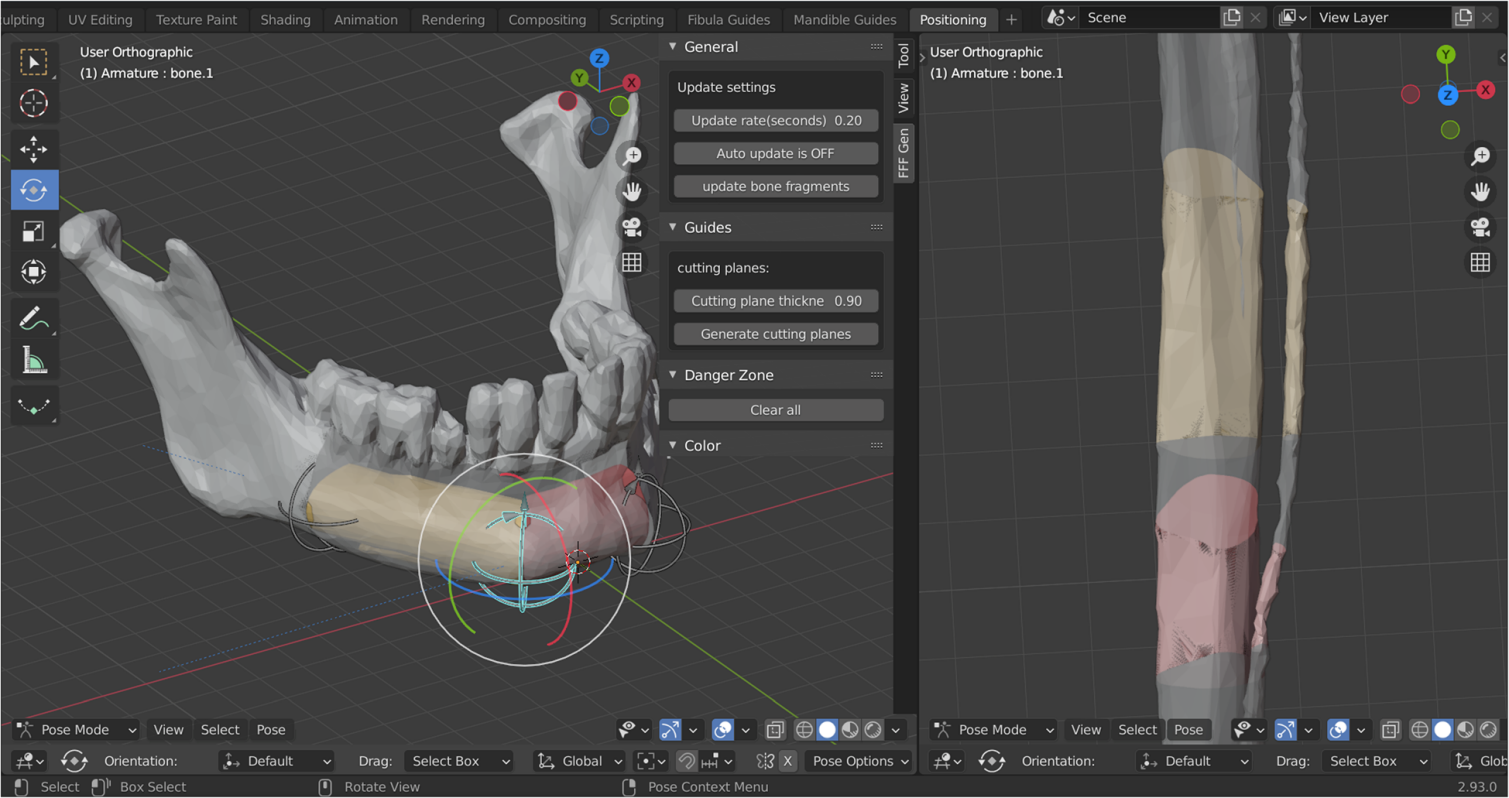
User interface after initialising the add-on, loading mandible and fibula three-dimensional models, and initialising the rig. Fibula grafts are virtually positioned according to the native mandible

The workflow can be split into several main steps. The first step is to initialise the add-on, which is done by selecting the appropriate option in the user interface. This will change Blender’s user interface providing the user with additional options as shown in Fig. 2. After fibula and mandible files (models exported from the image segmentation software) are imported, the user must manually check and correct their orientation and dimension. This is required as some 3D model formats do not store scale/unit information and errors can occur (for example, incorrect conversion from inches to millimetres). Fibula long axis should be aligned with the *y*-axis, with the posterior side facing the positive direction of the *y*-axis. The bottom of the mandible should be aligned with the *x*-*y* plane, with the front of the mandible aligned with the positive direction of the *y*-axis in 3D space. The user must specify the desired number of grafts. The software will set up all required Boolean operations and constraints automatically, using an internal object structure and naming scheme.

Users then proceed to the graft positioning step as shown in Fig. 2. The user interface is split into two areas. In the left area, the users fit the fibula grafts to the mandible. The area on the right shows how the grafts fit the fibula. Boolean operations are used to mask out parts of the mandible and fibula objects to aid in graft positioning. The grafts are positioned by translating and rotating the control objects (Fig. 2) whose initial positions are determined using predefined variables. While positioning the grafts, users should also consider the location of perforating vessels. The grafts should be positioned in such a way that the perforating vessels remain in the harvested segment. If the CT angiography of the lower legs was not performed or if the perforating vessels are not clearly visible in the model, Blender provides measuring and annotating tools that can be used to mark the approximate position of the perforating vessels (usually 9 cm from the fibular malleolus, in cranial direction).

Once the grafts have been positioned, cutting planes are generated based on the user-defined thickness and the location, rotation and shape of the grafts. They are automatically offset to compensate for the loss of material that occurs during the osteotomy.

**Fig. 3:**
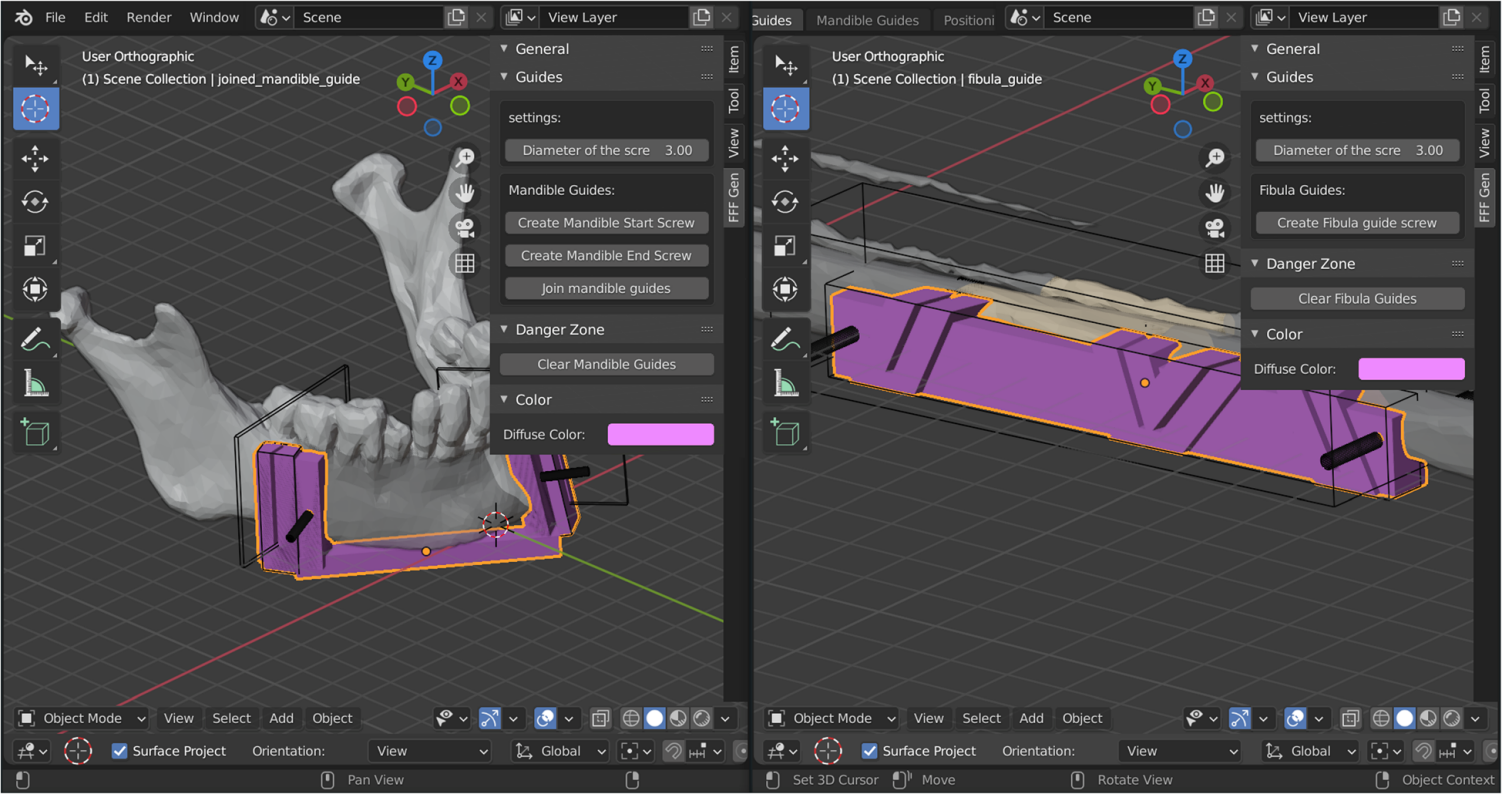
Generation of the fibula and mandible cutting guide models

Both fibular and mandibular guides are generated by using a set of Boolean operations on predefined objects. The initial positions and dimensions are determined by predefined values and locations of the grafts from the previous step. The guide modelling process does not require direct mesh manipulation. The guides are customised by performing rotate, translate and scale operations on the generated predefined objects (Fig. 3). Users have the option of generating screw holes that can be independently positioned and rotated. It is suggested that the diameter of the screw holes is slightly larger (0.1–0.2 mm) than the screws themselves in order to compensate for tolerances during 3D printing.

## Case studies

After the hospital’s Ethical Committee’s approval (IRB number: R2 – 6974/2020, approval date June 9, 2020) and until the publication of this article, our team at the University Hospital Osijek performed five successful mandibular reconstruction procedures. Personalised 3D printed osteotomy guides were designed using the developed tools. All four patients have given their written informed consent for surgery, as well as permission to use their diagnostic imaging material for the production of the personalised cutting guides and publication of the paper. Mandibular and fibular osteotomy guides were 3D printed with Stratasys Objet30 (Stratasys Ltd., Eden Prairie, Minnesota, USA/Rehovot, Israel) using Stratasys VeroGlaze MED620 biocompatible material. Prior to the surgery, osteotomy guides were sterilised using hydrogen peroxide plasma.

**Fig. 4:**
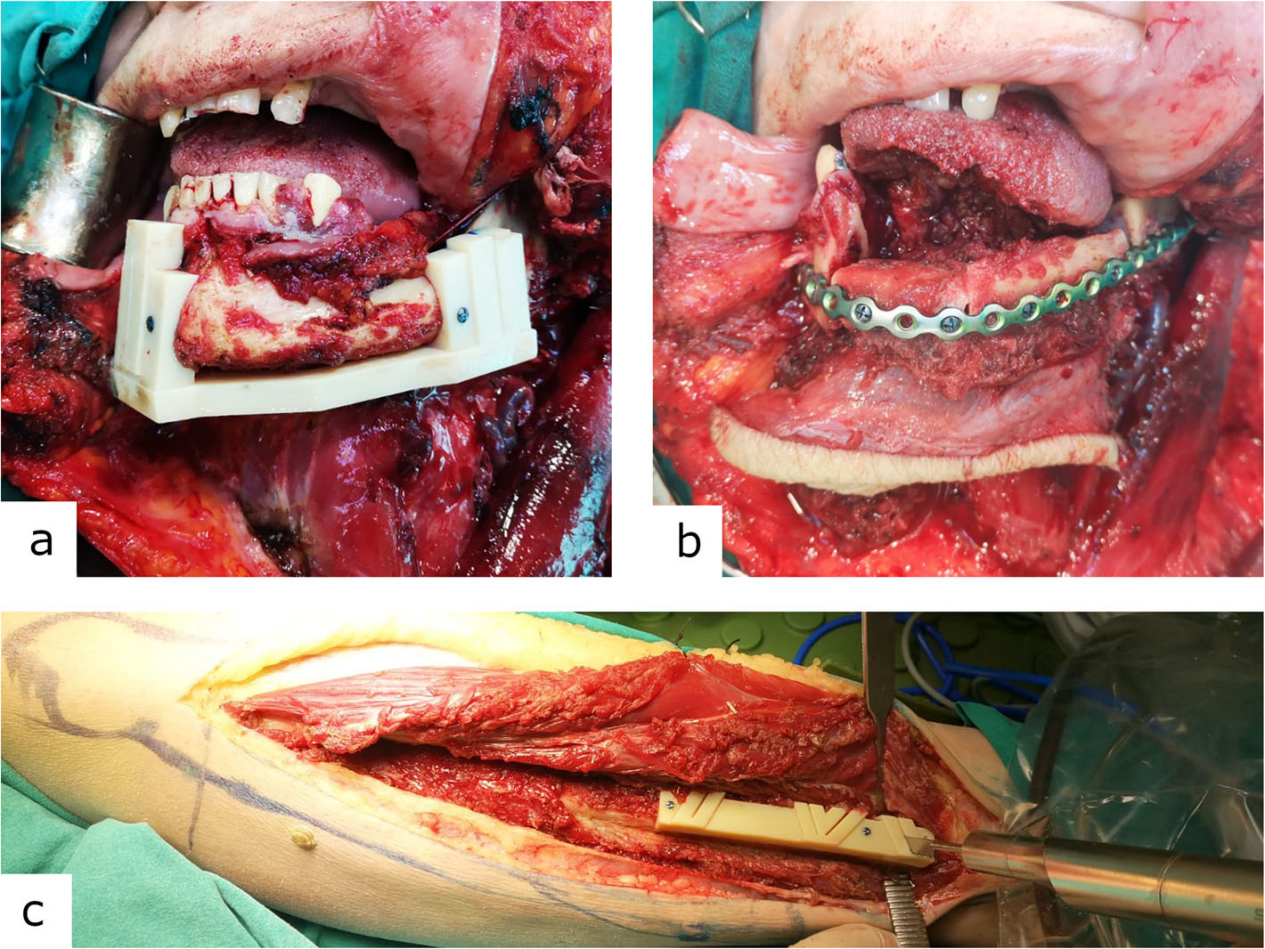
**a** Properly positioned and perfectly fitting three-dimensional printed cutting guide for mandible osteotomy. **b** Well-positioned bony grafts fixed with pre-shaped reconstruction plate. **c** Properly positioned three-dimensional printed personalised cutting guide for fibula osteotomy

The first patient had a small right-sided mandibular body defect. One bone segment graft was harvested from the right fibula which required two osteotomies. The second patient had a larger right-sided mandibular body defect. The third patient had a left-sided mandibular defect as seen in Figure 4. The fourth patient had a defect in mental protuberance. Two bony grafts with four osteotomies were used in the second, third and fourth cases.

On follow-up examinations, patients did not develop any late complications. Both, donor site and reconstructed mandibula healed properly with satisfactory symmetry of the lower jaw facial lines.

## Discussion

Surgical resection and reconstruction were based on surgeons’ experience, and the final decision was made intraoperatively. With the introduction of 3D printing into surgeon’s daily routine, they had the opportunity to perform better preoperative planning and go into the operating room with a clear idea of the extent of tumour resection, size and shape of the fibular bony fragments and fitting osteotomies. Restoring facial contour after tumour resection is one of the main goals after ablative surgery, and fitting osteotomies is integral in the process. Without cutting guides, osteotomies would be decided in the operating room making the work less precise and more time-consuming [2].

Scanning parameters of the lower legs CTA, described in the “Data acquisition and 3D model generation” section, proved to be satisfactory for a good peroneal artery perforating branches delineation, as seen in Fig 1.

Unlike the described workflow by Dell’Aversana Orabona et al. [7], Ganry et al. [8] and Numajiri et al. [9], where measurements of the distances, angles and all 3D modelling are mostly done manually, our approach provides a framework where surgical guides can be produced using a set of relatively simple 3D operations (a combination of translating, rotating and scaling objects in 3D space). The more advanced operations, such as parenting and setting up Boolean operations, osteotomy plane angulation, etc. are hidden behind newly implemented operators. In four successful cases, our technique proved to be safe for clinical use. It provides a faster workflow, as cutting-guide modelling time is reduced to 15 min or less compared to other authors who report times of 50 min or higher [7, 8]. The method is simplified so the end-users (surgeon, radiologist or radiology technician) do not need to be proficient in 3D CAD/CAM software as many hospitals still do not employ biomechanical engineers. Additionally, as changes in graft dimensions are observed in real-time, this framework also serves as a tool for “virtual surgery” and visualisation of the resulting guides and fibula grafts.

## Data Availability

The latest version of the Add-On can be downloaded from the following GitHub repository: https://github.com/lsimic/FFFGenBlender The version of the Add-On used in this article can be found in the following link: https://github.com/lsimic/FFFGenBlender/releases/tag/2.83_LTS Video tutorial: https://youtu.be/DA6HoQ-Avss Links to download the software used—Blender (note, version 2.83 LTS branch was used): https://download.blender.org/release/Blender2.83/ Links to download the software used—3D slicer: https://download.slicer.org/

## Abbreviations

3D: Three-dimensional
CAD: Computer-aided design
CAM: Computer-aided manufacturing
CT: Computed tomography
DICOM: Digital Imaging and Communications in Medicine
PACS: Picture archiving and communication system
ROI: Region of interest
